# Sulfidation-Oxidation Resistance of Thermal Diffusion Multi-Layered Coatings on Steels

**DOI:** 10.3390/ma14195724

**Published:** 2021-09-30

**Authors:** Tomasz P. Dudziak, Ewa Rząd, Eugene Medvedovski, Gerardo Leal Mendoza

**Affiliations:** 1 Centre of Materials Research, Lukasiewicz Research Network, Krakow Institute of Technology (Former Lukasiewicz—Foundry Research Institute), 73 Zakopianska St., 30-418 Krakow, Poland; ewa.rzad@kit.lukasiewicz.gov.pl; 2 Endurance Technologies Inc., 71-4511 Glenmore Trail SE, Calgary, AB T2C 2R9, Canada; eugenem@endurancetechnologies.com (E.M.); gerardom@endurancetechnologies.com (G.L.M.)

**Keywords:** thermal diffusion coatings, aluminides, borides, steel, sulfidation-oxidation resistance

## Abstract

The high-temperature sulfidation-oxidation corrosion resistance of protective coatings deposited on carbon and 316L steels was studied. The coatings obtained via the thermal diffusion process had multi-layered architectures and consisted of aluminides, iron borides, or iron boride–TiO_2_ layers. The protective coatings experienced a minimal rate of mass changes, insignificant scale formation, and no delamination and surface micro-cracking after 504 h of exposure in 1% (Vol.) H_2_S-air atmosphere at 500 °C. Furthermore, the coatings demonstrated a high degree of integrity compared to bare 316L stainless steel. Aluminized steels demonstrated the highest performance among the studied materials. The developed thermal diffusion coatings are promising candidates due to their enhanced stability in H_2_S–air atmosphere; they may be employed for protection of inner and outer surfaces of long tubing and complex shape components.

## 1. Introduction

Intensive gaseous corrosion of steels and alloys in combustion processes, especially in the presence of S- and H_2_S-containing gases, results in accelerated destruction and failure of tubing and other components made of steels and alloys, leading to unpredictable equipment shutdowns, costly maintenance, and increased production cost. Since the corrosive sulfidation atmosphere may vary from highly reducing to oxidizing, the corrosion impact of H_2_S and other gases on metallic components, e.g., boiler tubes, superheaters and reheaters, and others, may differentiate [1,2]. The corrosion rate in H_2_S-containing gaseous environments is related to dissociation of H_2_S to hydrogen and sulfur at rather low temperatures (below 500 °C) and consequent interaction of metals with S resulting in the metal sulfide scale formation. The occurring metal sulfides are not very stable due to their low free energy values [3,4]. The formed scales are rather porous and poorly adhere to steels and alloys, and, as a result, they have a lower protection rate in comparison to oxide scales [4,5,6,7,8]. These features lead to rapid diffusion of S and H_2_ through porous scales, lattice defects, and hydrogen cluster generation, with consequent interaction of H_2_ and S with the base metals significantly increasing corrosion and embrittlement and, hence, further disintegration of steel components [3,4,9]. In the sulfidation-oxidation conditions, e.g., when air volumes in the gas flows are substantial, sulfur occurring due to H_2_S decomposition can easily oxidize with sulfur dioxide (SO_2_) formation. Due to the reaction of H_2_S with oxygen, sulfur or sulfur dioxide can also occur. Although the interaction of H_2_S with SO_2_ generally occurs at high temperatures (greater than 1000 °C) with formation of elemental sulfur, the reaction temperature can drop significantly, even to below 500 °C, in the presence of certain metallic or oxide catalysts. The elements, which may be contained in some steels and alloys employed in power generation units, and their oxides can work as catalysts promoting the mentioned reactions.

Relatively inexpensive carbon steels and low-alloy steels, mostly used for tubing in power generation, have rather poor performances in high-temperature (above 500 °C) oxidation, sulfidation, or sulfidation-oxidation conditions. It is related to their rapid oxidation with the formation of detachable iron oxide scales (in case of oxidation environments) or formation of porous iron sulfide scales (FeS_x_), which are easily peeled off, as well as surface cracking [10,11,12]. Stainless steels and even more expensive Ni-based and Ti-based alloys also experience corrosion issues due to detachment of the sulfide scales and surface micro-cracking at service temperatures (350–500 °C), although to a lesser extent [4,6,13,14,15,16,17]. The addition of Al and refractory metals to stainless steels’ and Ni-based alloys’ compositions promotes their sulfidation resistance [17,18,19,20]. However, these routes lead to a further cost increase of expensive steels and alloys.

Surface engineering, specifically through the advanced coating formation route, can be considered as an alternative strategy for high-temperature sulfidation and sulfidation-oxidation protection, especially if low-cost steels are used as substrate materials. Different coating materials and technologies for corrosion protection can be listed [21,22,23,24,25,26]. However, many coating options either have poor performance at high temperatures or insignificant thicknesses compared to possible structural defects, or poor adhesion and bonding to steel substrates, which leads to detachment issues at elevated temperatures. Some reliable coatings are inapplicable for protection of the inner surface of long tubing and complex shape components. In contrast, the thermal diffusion surface engineering technology, specifically boronizing and aluminizing of steels, can be used to protect critical components, including long tubing, exposed to harsh corrosion conditions [27,28,29,30,31,32,33,34,35,36,37,38]. Recently, advanced coating materials obtained through the thermal diffusion process were considered and tested, for the first time, for high-temperature sulfidation applications with promising results after exposure in H_2_S-Ar flows at 500 °C [39]. In this work, the same materials were exposed to a H_2_S-air atmosphere at 500 °C to compare their behavior in two different H_2_S-containing atmospheres and to evaluate the influence of atmosphere on corrosion resistance. This study should enhance the understanding of materials’ performance in high-temperature corrosion and assist materials’ selection specifically for industrial applications.

## 2. Experimental

### 2.1. Materials and Process

Austenitic steel 316L was selected as the baseline material for the sulfidation-oxidation testing. This steel was selected as a typical material with high corrosion resistance for boiler tubes, superheaters, reheaters, and other components in coal-fired plants, Integrated Gasification Combined Cycle (IGCC), and many other combustion and refinery industrial processes if enhanced performance is required. This steel, as well as carbon steel A36/44W (its composition is identical to carbon steels widely used for tubing), was also used as the substrate material for the thermal diffusion processing. The chemical compositions of these base materials, according to the Mill Test Reports (MTRs), are shown in Table 1. The test coupons were cut from steel bars to dimensions of ~25 × (13.5−14) × 6.35 mm (~1”× 0.5” × 0.25”); a hole of ~6.5–7.0 mm was drilled near one end of each coupon. All coupons were finished to remove sharp edges and corners, then blasted with aluminum oxide grits, and washed with acetone to remove surface scale and grease.

The test coatings were prepared according to the proprietary thermal diffusion technology established by Endurance Technologies Inc. (ETI) in Calgary, Canada. The coating process was described in detail in previous works [29,30,35,37,39]. The list of materials for sulfidation-oxidation testing is shown in Table 2. It should be noted that bare carbon steel was not included in the study since carbon and low-alloy steels have poor performance in sulfidation conditions [12] and are not reliable for industrial high-temperature corrosion applications.

### 2.2. Sulfidation-Oxidation Testing

**Figure 1:**
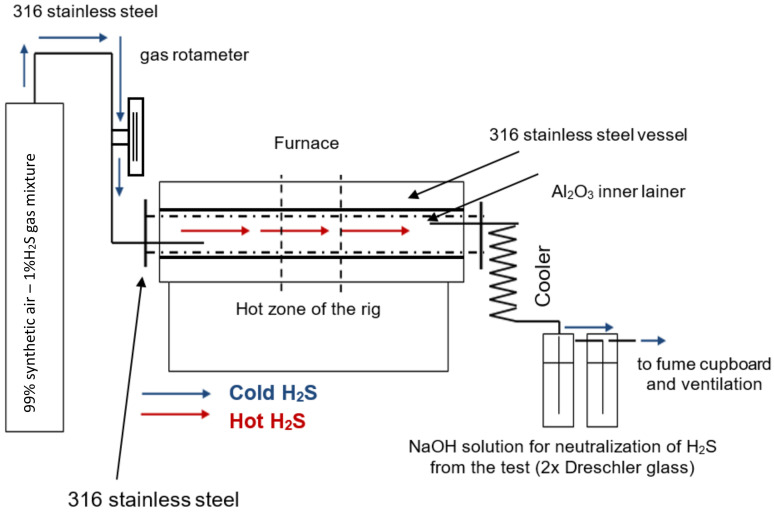
Schematic of the experimental rig for high-temperature sulfidation-oxidation.

The testing was conducted in the 1% (Vol.) H_2_S-air gas flow (50 Nml/min); the H_2_S concentration in the gas mixture was selected according to the general assumption that H_2_S contents in coal-fired boiler combustion gases are below or approximately 1 Vol.% [12,39]. The gas mixture was provided by Air Products. The experimental procedure was the same as reported in the paper published earlier [39]. The experimental set-up used in this work is shown in Figure 1.

Similar to our previous work, the samples placed onto the high-alumina refractory holder maintaining adequate gas flow circulation were inserted into the furnace chamber. The samples were heated to 500 °C with a ramp rate 5 °C/min and then held at this temperature for a certain time. The testing comprised 3 cycles of 168 h (1 week) per cycle. After each cycle, the samples were examined according to the previous study [39].

### 2.3. Examination

Each sample was measured with an electronic micrometer for the surface area calculation, cleaned in the ultrasonic bath at ~40 °C for 15 min, and weighed before and after each high-temperature test cycle using an electronic balance SARTORIUS CPA-225D, Goettingen, Germany (accuracy of 0.01 mg). The specific mass change was calculated as *Δm/S*, where *Δm* is the mass change [mg] and *S* is the surface area of the sample [cm^2^]. The exposed samples were pictured using a single lens DSLR camera Canon EOS 70D (Japan, Tokyo) coupled with a Canon MP-E 65 mm f/2,8 macro lens. The coating–substrate adhesion was checked by scratching using a steel knife. In order to evaluate the degradation process of the exposed samples, the structural examinations with a light optical microscope (LOM) MEIJI Techno 1M7200 (Japan, Saitama) (for materials’ cross-sections) and with scanning electron microscope (SEM) JEOL JSM-IT300LV (Japan, Tokyo) combined with X-ray energy dispersive spectrum analysis (EDS)—JEOL JED-2300 DRY SDD EDS detector (for materials’ surfaces) were carried out. The details of the cross-section’s preparation were reported in previous works [35,37,39]. The materials’ micro-hardness was determined according to ASTM E384-17 using the micro-tester Clark Instruments CM 400AT (Novi, MI, USA); the testing was conducted using a Knoop diamond pyramid at the 100 g indentation load (HK0.1). To generate average values of the micro-hardness results, at least 10 indentations were applied to each cross-section of the polished sample under the hardness tester’s microscope.

## 3. Results and Discussion

### 3.1. Coatings’ Structure and Compositions

**Figure 2:**
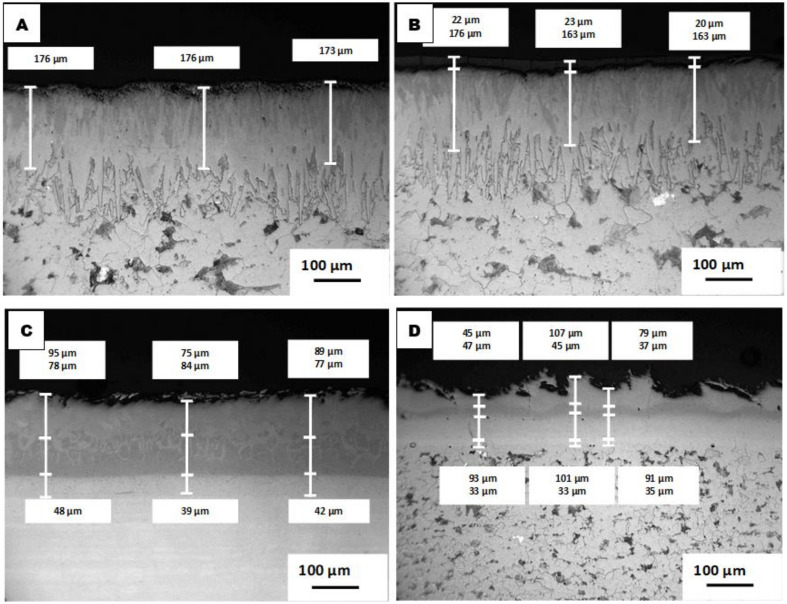
Microstructure of the coatings (LOM, cross-sections) before testing: Top layer—(**A**) boronized carbon steel (B) (left); boronized carbon steel with (**B)** TiO_2_ top layer (BT) (right). Bottom layer—(**C**) aluminized stainless steel 316L (6A) (left); (**D**) aluminized carbon steel (A) (right).

The obtained thermal diffusion coatings have multi-layered architectures where the coatings consist of two or more layers that occurred through a single manufacturing (heat treatment) step. The coatings consist of either boride or aluminide layers for boronized and aluminized steels, respectively. One of the major features of the coatings’ formation is the inward diffusion of B or Al to the steel structure and the outward diffusion of Fe and other steel constituents (e.g., Cr and Ni from stainless steel) and consequent formation and growth of crystalline iron borides or iron aluminides on the steel surface. All the obtained layers are well-consolidated with minimal porosity and with no interface between the layers and the steel substrates. The details of the thermal diffusion process and the boride- or aluminide-based coatings’ formation were described elsewhere [28,29,30,31,32,35,39]. The boronized steel structure (materials B and BT) comprises two layers, e.g., the inner layer consisting of the Fe_2_B phase covering the carbon steel substrate and the outer layer consisting of the FeB phase (Figure 2). Both well-consolidated layers have identical “saw-tooth” morphology. The features of the boronized steel structure and compositions were described earlier [29,30,35]. The total coating thickness (case depth) is approximately 170–175 µm (~0.007”). It was determined from the top of the coating to the approximate middle of the “teeth”. For the boronized steel covered by an additional TiO_2_ layer (materials BT), this top layer has a thickness of ~20 µm; however, some discontinuity in this layer and its partial peeling-off are observed. This peeling-off issue is related to the imperfectness of the TiO_2_ layer applied and that this layer became thicker than required; however, despite this issue, the nano-sized TiO_2_ particles easily penetrated the FeB surface asperities.

The aluminized samples have three to four layers (Figure 2), where each layer differentiates in Al contents and contains different aluminides according to the diffusion-induced process [28,35,36,37,39]. Thus, the transition layer (the first layer covering the steel substrate) mostly consists of the Me_3_Al (e.g., Fe_3_Al) phase, while the next (main) layer mostly consists of FeAl (for the carbon steel substrate) or Fe(Cr,Ni)Al (for the stainless steel substrate). The top Al-rich layers consist of aluminides with higher Al contents, such as Me_2_Al_5_. Accordingly, the Al contents in different layers varied from ~3–7 wt.% for the transition layer to ~43–48 wt.% for the Al-rich layers. The thickness of each layer differentiates depending on the steel substrate and the layer composition. The total thicknesses of the crystalline aluminide-based coatings are ~200–225 µm (0.008–0.009). The aluminide coating on stainless steel is significantly smoother and more even compared to the coating on carbon steel, as clearly seen in Figure 2.

### 3.2. Materials’ Appearance

**Figure 3:**
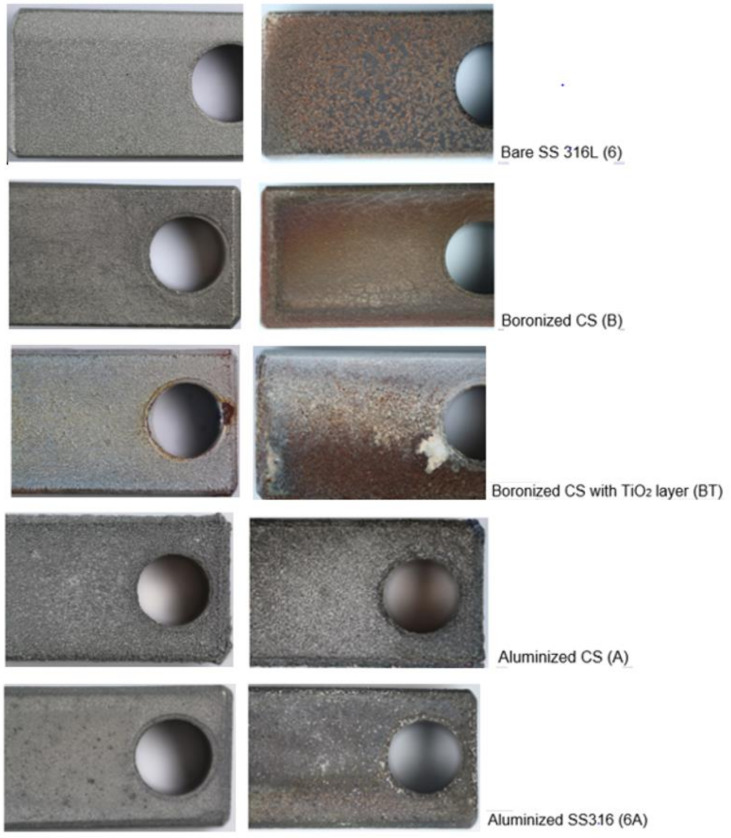
Appearance of the samples prior to corrosion testing (**left**) and after sulfidation-oxidation (H_2_S-air, 500 °C) for 504 h (**right**).

The samples with diffusion-induced coatings showed a high degree of protection demonstrating no spallation, flaking, or deterioration under sulfidation-oxidation exposure (Figure 3); however, some peeling-off of the top TiO_2_ layer was observed mostly near the drilled hole, i.e., on the corners with an elevated stress concentration. The observed color change was spotted for the bare stainless steel (samples 6) and boronized steel (samples B and BT) with a slightly stronger brownish color after the longer exposure, while the color of aluminized steels remained on the original level. However, the color deviation could be only superficial, particularly for the samples with the coatings, since no visual scale formation was detected. The scratching with a steel knife confirmed the absence of the generally soft sulfide scale and the presence of hard coatings (harder than a steel knife).

The boronized samples became even smoother with some sort of the surface glassifying, especially after the third week of exposure. In contrast, the uncoated 316L steel experienced a high degree of external scale formation and degradation due to the scale detachment (the scale was partially peeled-off at handling, especially at scratching with a knife), similar to previous results on testing in H_2_S-Ar [39]. Additionally, small pits on the steel surface could be observed under string light, especially using a magnified lens. Because of this, the formed rather soft and detachable scale on the bare stainless steel cannot be considered as protective, similar to sulfidation conditions in H_2_S-Ar [39]. Comparing the present results and previous data recorded in [39], the scale formation on bare steel was less in the case of H_2_S-air exposure compared the H_2_S-Ar. The sulfide scale was also denser with a better adhesion to steel. It was expected because of the higher oxygen content in the sulfidation-oxidation gas mixture. Higher oxidation in the H_2_S-air flow also promoted, as noted above, a greater extent of surface glassifying for the boronized samples.

### 3.3. Kinetic Data

**Figure 4:**
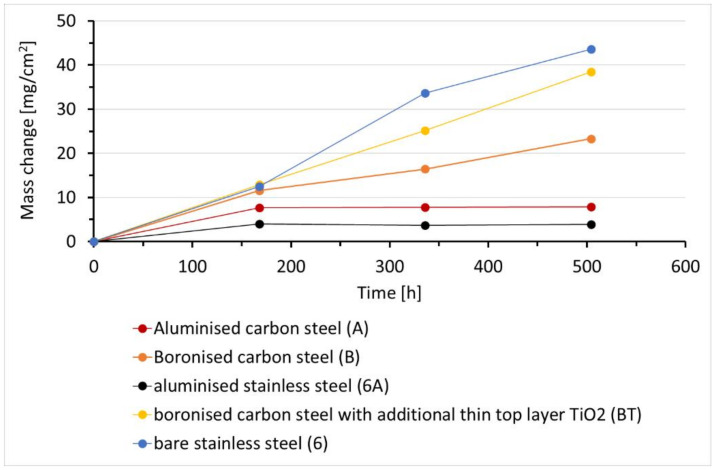
Specific mass change vs. time (kinetic curves) for the studied samples after sulfidation-oxidation in H_2_S-air at 500 °C for 504 h.

The kinetic curves of the exposed samples in H_2_S-air conditions are shown in Figure 4. Similar to the sulfidation test in the H_2_S-Ar atmosphere, the highest mass gain after exposure in H_2_S-air atmosphere was recorded for the uncoated stainless steel 316L (samples 6) with a gradual increase with the exposure time due to the formation and growth of the scale consisting of sulfides and oxides. In comparison to the H_2_S–Ar conditions, the mass gain of the bare 316L steel samples increased from ~5 mg/cm^2^ to almost 45 mg/cm^2^ after 3 weeks of exposure in the H_2_S-air flow. This difference accounts for the combination of sulfidation and oxidation processes of the uncoated stainless steel instead of only the sulfidation process. It is assumed that, because the Gibbs free energy formation for oxides is more negative than that for sulfides [40], the oxide-based scale formation is more favorable than sulfides.

Due to partial detachment of the rather soft scale on the steel surface, the kinetic data recorded cannot be considered as very accurate (two concurrent issues occurred: the mass gain due to scale formation and the mass loss due to the scale partial peeling-off). The tendency in the mass gain of the B and BT samples is also similar to that observed in the previous work [39], where the BT samples showed a slightly higher mass gain than the B samples. Since no visible soft sulfide scales were observed on the boronized samples, the mass gain for these samples may be attributed mostly to the iron boride surface oxidation and the surface glassifying. Partial detachment of TiO_2_ in some areas of the BT samples also does not make the recorded kinetic data very accurate. The samples with aluminized coatings experienced the lowest mass gains, with similar results obtained for these materials when they were exposed to the H_2_S-Ar flow. For these coatings, the plateau on the kinetic curves was reached already after the first week of exposure, and the recorded kinetic data are significantly more reliable compared to other tested materials. The mass gain for the samples A (aluminized carbon steel) is slightly higher than of the samples 6A (aluminized stainless steel), and it can be explained by the higher surface roughness of the former materials, as can be seen in Figure 2. The outcomes in this work clearly show that the aluminized coating developed a protective oxide scale. In this work, *k_p_* values were not calculated not only due to the rather short test duration (i.e., small numbers of test data), but also due to the rather approximate data for some materials, particularly bare stainless steel and BT samples, where the formed sulfide scale or the TiO_2_ layer were partially peeled-off.

### 3.4. SEM-EDS Surface Analysis

**Figure 5:**
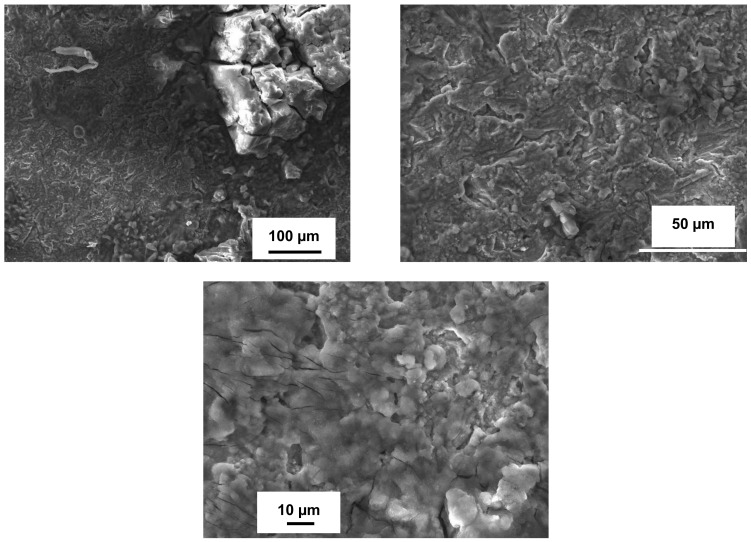
SEM surface morphology of stainless steel 316L after 504 h of exposure in H_2_S-air at 500 °C.

**Figure 6:**
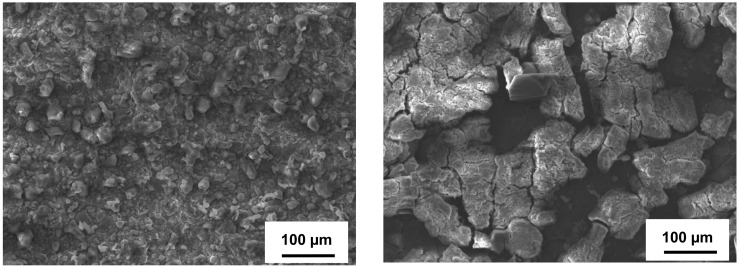
SEM surface morphology of carbon steel with boronized coatings after 504 h of exposure in H_2_S-air at 500 °C, Left—boronized steel (B), Right—boronized steel with a top TiO_2_ layer (BT).

**Figure 7:**
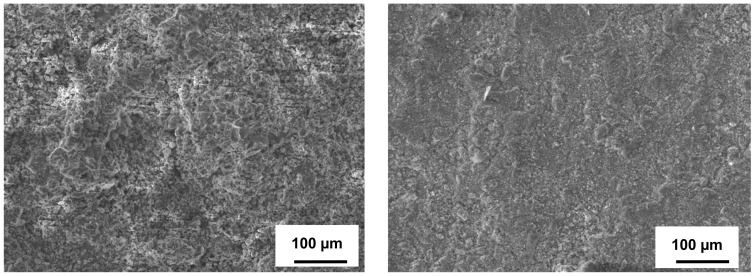
SEM surface morphology of steels with aluminized coatings after 504 h of exposure in H_2_S-air at 500 °C. Left—aluminized carbon steel (A), Right—aluminized stainless steel (6A).

The SEM images of the samples’ surfaces after exposure in H_2_S–air for 504 h (three test cycles) are shown in Figure 5, Figure 6 and Figure 7. Bare stainless steel after high-temperature sulfidation-oxidation had a rough and uneven scale (Figure 5), although this scale is denser compared to the scale that occurred after sulfidation [39]. The scale was partially peeled-off where some top scale “islands” remained on the surface of the “underneath” scale. Some cracks can be observed on the surface. These cracks may be related to cooling from the high temperature when tensile stresses are greater than compressive stresses within the scale and at the scale–steel interface. The summarized EDS surface analysis data (Table 3) performed on the scale in different places detected mostly Fe (23–28 wt.%), O (45–53 wt.%), S (8–16 wt.%), as well as Cr (from <1 to 12 wt.%), and Ni (3–5 wt.%), and these data confirm an uneven scale structure and composition. Detailed observation of 316L steel indicates that the top scale layer consisted of significantly higher contents of O and S (reaching up to 50–53 and 14–16 wt.%, respectively) and lower concentrations of Cr (0.25–0.7 wt.%), Ni (3–3.5 wt.%), and Fe (20–23 wt.%) than the layer localized underneath with Cr (11–12 wt.%), Ni (5–6 wt.%), and Fe (26–28 wt.%). These data clearly show that the 316L steel experienced sulfidation–oxidation’s impact with the surface deterioration.

During sulfidation-oxidation, several simultaneous reactions take place at elevated temperatures (e.g., at 500 °C), affecting the 316L steel surface. Nevertheless, we assume that the activity of H_2_S, S, and some other S-containing gases in the gaseous environment is lower than the oxygen activity since the S/O_2_ ratio is shifted towards O_2_. According to the EDS analysis results and analyzing possible high-temperature reactions between steel constituents and the gaseous phase containing H_2_S, S, O_2_, and SO_2_, the following reactions may be assumed in Table 4:

The high-temperature reactions with “minor” elements from 316L steel (e.g., Mn, Mo, Cu) may also occur in these conditions. The presented reactions do not occur at the same time due to the higher concentration of O_2_ in the gas mixture and greater free energy formation (−ΔG) for oxides compared to sulfides [40]. Thus, as we stated above, the formation of oxides may be more preferential than sulfides, although, according to the SEM-EDS analysis, the presence of sulfides is also evident. The formation of Fe_2_O_3_ may also take place due to oxidation of Fe_3_O_4_, while the FeO phase is unstable, and this phase formation is unexpected. However, during the process, this unstable oxide may react with SO_2_ gas forming iron sulfite:
FeO + SO_2_ → FeSO_3_                  20

As opposed to bare stainless steel, the surface of boronized carbon steel (samples B) was rather even with insignificant scale presence after the exposure in H_2_S–air flow at 500 °C after 504 h (Figure 6). No blisters, specific soft and rough iron sulfide scale, or peeling-off were observed. According to the summarized EDS analysis data (see Table 3), remarkable amounts of O, as well as the presence of B and Fe, were recorded, which indicates iron boride surface oxidation. The presence of S (reached to ~9–10 wt.%.) was also detected on the samples’ surface. Tiny spherical particles observed under SEM may be related to S_2_, which deposited onto and adhered to the (FeB)_x_O_y_ surface, especially because the iron boride surface originally had some roughness. In the previous work [39] where the samples were subjected to a H_2_S-Ar environment, the formation of surface micro-cracks and a thin boron sulfide scale were observed on the boronized steel surface; however, in the present study, a boron sulfide phase and notable surface micro-cracking were not observed. Furthermore, a smoother boronized surface indicates a “positive” influence of oxidation, promoting surface healing and glassifying.

The surface of boronized steel with a top TiO_2_ layer (BT sample) shown in Figure 6 had micro-cracks, particularly “fragments” containing micro-cracks. This issue may be related to the TiO_2_ top layer with an excessive thickness, which partially peeled-off during the samples’ preparation. Since the top TiO_2_ layer had numerous discontinuities, the EDS analysis detected remarkable amounts of Fe, B, and O (Table 3) on the surface, confirming the oxidation of the FeB phase. The EDS analysis also detected ~9–10 wt.% of S. Sulfur particles could deposit and adhere to the uneven surface of the BT sample, specifically penetrating and accumulating in the occurred micro-cracks and discontinuities in the TiO_2_ layer. These sulfur particles together with the formed (FeB)_x_O_y_ oxidation layer facilitate the top TiO_2_ surface micro-cracking and its partial detachment, leading to more surface discontinuity. However, the underneath FeB layer with the formed (FeB)_x_O_y_ was free of destruction, blisters, and other defects.

The aluminized steel surfaces were very homogeneous after the sulfidation-oxidation testing for 504 h, as shown in Figure 7. No cracks, blisters, chipping, or other defects, as wells as no sulfide grains, were observed. Only very occasional spherical particles were observed on their surfaces, which are probably related to sulfur (the EDS analysis in the local areas confirms this). According to Table 3, the S contents on the aluminized steels’ surfaces are below 2 wt.%. Furthermore, the S content is lower for samples 6A compared to samples A. These low S contents confirm no sulfide formation, and the very small S amounts may be attributed only to S particle adherence to the samples’ surfaces. The smoother surface and smaller S contents on the surface of 6A samples (even below 1 wt.%) compared to A samples is explained by the materials’ original morphologies, particularly by the rougher surface of the latter material. Significant contents of O together with Al and major constituents (Fe from carbon steel and Fe, Cr, Ni from stainless steel) observed on the aluminized surfaces confirm surface oxidation of the aluminides and the formation of an Al_2_O_3_-based skin. The Al concentration in the Al-rich layer, which can serve as a donor of Al, was high enough to form a thin but stable Al_2_O_3_-rich oxide skin. It is well known that Al_2_O_3_ is very stable at high temperatures due to high free energy formation (−ΔG) [28,31,40,41]. According to the conducted studies [41,42,43], at relatively low temperatures, amorphous Al_2_O_3_ occurs first, and then, under stable oxidizing conditions, it gradually transforms to the θ-Al_2_O_3_ phase and further to γ-Al_2_O_3_ with consequent consolidation. Only under stable and long oxidation conditions, especially at elevated temperatures, those metastable phases may further transform to stable α-Al_2_O_3_. Since the oxygen content in H_2_S-air is significantly higher compared to H_2_S-Ar, surface oxidation of the aluminides and related protective oxide skin formation are greater in the current testing.

### 3.5. Microstructure Analysis

**Figure 8:**
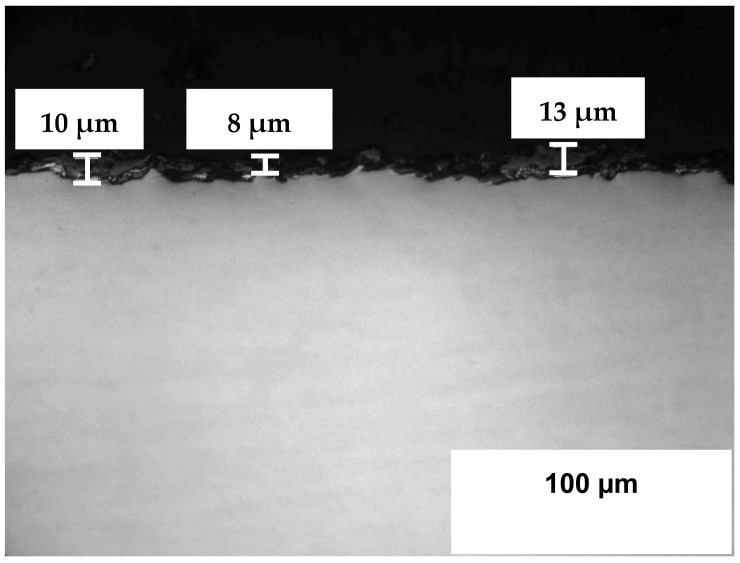
Structure of stainless steel 316L (6) after exposure in H_2_S-air at 500 °C for 504 h.

The materials’ microstructure examination using LOM for the samples’ cross-sections confirms the results from the SEM-EDS surface analysis and provides further understanding of the materials’ integrity. Uncoated 316L steel experienced formation of oxide-sulfide scale with a thickness of several microns, reaching 8–15 µm after 504 h (Figure 8). The thinner and denser scale that occurred in the present H_2_S-air study compared to the mostly sulfide scale, which occurred after H_2_S-Ar exposure, was attributed to the significant oxidation process. However, the scale peeling-off that occurred during handling and, especially during cross-sections’ preparation, made the cross-sections’ images less representative with a not very accurate estimation of the oxide-sulfide scale thickness.

**Figure 9:**
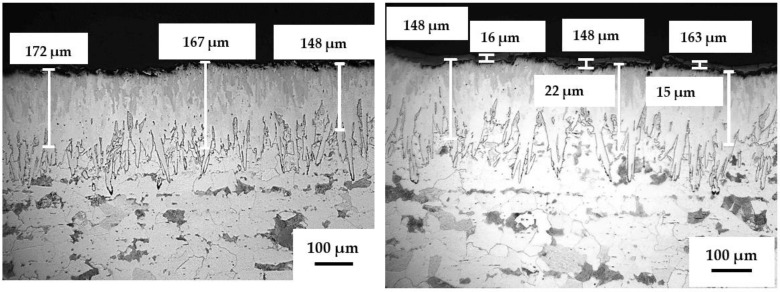
Microstructure of boronized carbon steel (B)—left, and boronized carbon steel with TiO_2_ top layer (BT)—right after exposure in H_2_S—air at 500 °C for 504 h.

The microstructure of boronized carbon steel (samples B) remained without visible changes after 504 h of sulfidation-oxidation (Figure 9). The same double-layer coating structure was observed; however, the coating thickness became slightly smaller (~150–170 µm). No blisters and cracks within the coating structure, delamination, or peeling-off were found. A very thin scale was detected, which is related more to oxidation of iron boride than the sulfide formation. The probability of the glassified B-S-O or Fe-B-S-O film formation on the FeB surface could also be assumed, according to Schrooten et al. [44], who described boron oxysulfide glasses. Similar results were obtained for the boronized carbon steel with an additional top TiO_2_ layer (BT samples), with no changes in the double-layer iron boride structure and no blisters, cracks, or any other internal defects (Figure 9). The TiO_2_ top layer thickness also remained unchanged (~12–20 µm); however, it cracked and partially peeled-off. This issue with the top layer occurred, as was mentioned above, because this layer was originally thicker than required and was not very even in thickness. However, comparing the integrity of the TiO_2_ layer in the present work and from the previous work [39], the TiO_2_ layer observed after exposure in the H_2_S–air flow is denser with less cracking and detachment issues. The sufficient amount of oxygen in the H_2_S-air atmosphere positively affects the coating integrity, reducing the discontinuity occurrence.

**Figure 10:**
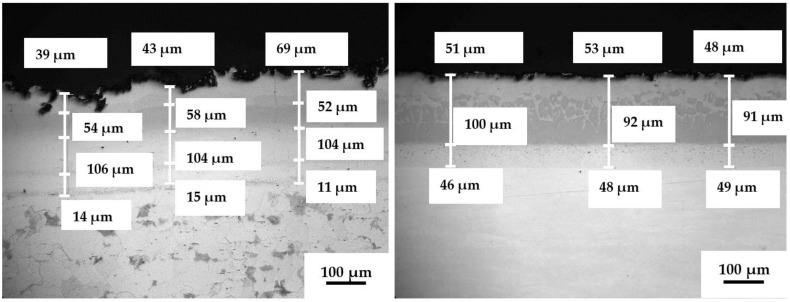
Microstructure of aluminized carbon steel (A)—left, and aluminized stainless steel 316L (6A)—right after exposure in H_2_S—air at 500 °C for 504 h.

The architecture of aluminized carbon steel and stainless steel after sulfidation-oxidation testing was also unchanged, as shown in Figure 10. Again, no blistering, cracking, or delamination within the coatings were observed. Slight micro-cracking and chipping in the top Al-rich layer in the samples of aluminized carbon steel can be explained by thermal stresses within the Al-rich aluminide layer and its elevated brittleness [28,35,38,41]. As noted above, the exposure in the oxygen-rich gas, despite the presence of H_2_S, promoted surface oxidation of the aluminides with formation of a thin, up to ~2.5–4 µm, protective layer. Although the surface of samples A and 6A contains Fe, Cr, Ni, Al, and O (Table 3), i.e., oxidized aluminides, it is suggested, according to published data [31,33,35,36,37,38,39,41], that the most external layer contains an extremely thin Al_2_O_3_ film. This film is well-adhered to the Al-rich aluminides, especially in the case of smoother 6A samples. Again, no visible formation of the specific sulfide scale was observed. Both aluminide and boride-based coatings provide complete protection of the steel substrates since no corrosion issues were observed for carbon steel and stainless-steel substrates.

### 3.6. Micro-Hardness Determination Results

The results presented in Table 5 clearly show that the micro-hardness values were not reduced for the coatings (both boronized and aluminized) after sulfidation-oxidation exposure. These results are the same as those obtained in our previous studies conducted in the H_2_S-Ar environment [39]. The obtained result clearly indicates a high structural integrity of the borides and aluminides, which the coatings are composed of. The same coatings’ hardness level confirms no or insignificant occurrence of surface micro-cracks, pits, or other structural defects, as well as a lack of transformation within the coatings’ constituents (e.g., their compositions) and new phase formation, since the materials’ hardness is defined, in a high extent, by the presence or absence of internal micro-defects. In contrast, bare 316L steel (samples 6) experienced some reduction of micro-hardness after high-temperature corrosion. This point is related to the steel structure weakening and scale formation on the unprotected steel surface.

### 3.7. Structural Coatings’ Features Affecting Corrosion Resistance

Since LOM and SEM-EDS examination did not reveal the S-based scaling on the boronized and aluminized surfaces, XRD analysis for the surfaces of these samples is not very reasonable. Based on the recorded data, we can make some general clarifications of the proposed materials’ corrosion resistance. The increased corrosion resistance of the boronized and aluminized steels obtained through the thermal diffusion technology is defined by the combination of the coatings’ composition and their multi-layered architecture. The coatings consist of either metal borides or aluminides, both with high crystalline lattice energies and strong and short covalent Me-B and Me-Al bonds [45,46,47,48,49]. The initiation of the reactions occurring in high-temperature corrosion is very unlikely for these compounds compared to bare steels, where the lattice energy is lower and metallic bonds are significantly weaker, i.e., these reactions may be initiated at significantly higher temperatures above service temperatures. Since, in contrast to bare steels, borides and aluminides do not contain “free” Fe (Fe is bonded with either B or Al), the sulfide formation is inhibited. As a sequence, the high-temperature H_2_S dissociation preferentially occurring on the sulfide surface [3,6] should also be delayed, which, in turn, should reduce the corrosion issues. The obtained materials have at least two layers, where the top inert layer protects the underneath inert layer. All the layers (either boride or aluminide-based) are well-consolidated with minimal internal micro-defects and with no visible delamination, cracks and other defects at the layers’ interfaces, with diffusion-induced bonding between the layers. The total coating thicknesses in the studied materials are significantly greater than 100 µm (as can be seen in Figure 3, ~170 µm or greater), and they did not change or changed insignificantly upon testing. This thickness is large enough to reduce the propagation of the corrosive environments to the steel substrate and provide effective steel protection. In general, the corrosion evolution in solid and dense chemically inert compounds occurs through micro-cracks and micro-defects from the surface to the middle of materials [21,50,51]. Each layer (well-consolidated and consisting of inert compounds) should be considered as protective. Because of this and due to the purposely produced multi-layered structure with diffusion-induced bonding between the layers and the substrates, the crack propagation, which is responsible for corrosion evolution, would be minimized. The micro-hardness data before and after testing confirm the high integrity of the proposed coatings. The features of the positive effect of the multi-layered structures on corrosion protection were considered elsewhere [30,35,37]. In addition, a ductile steel substrate supports hard and brittle multi-layered structures, serving as a “cushion” and promoting reduced micro-crack propagation. An additional top layer applied over the boronized steel should provide further protection if this layer consists of chemically inert materials. Thus, the TiO_2_ thin layer inhibits iron boride oxidation and sulfidation, particularly if this layer is of sub-micron or a few micron thickness with higher adhesion to iron borides. However, because of its excessive thickness and related micro-cracking and its partial peeling-off, its effectiveness was significantly reduced. Therefore, the top layer formation process should be optimized to minimize its thickness and to reach better adhesion and higher homogeneity. The thin alumina film that occurred on the aluminized steels in high-temperature sulfidation-oxidation conditions can also be considered as an additional protective layer since it is near crack-free and has good adhesion to the Al-rich aluminide layer. Al_2_O_3_ is significantly more thermodynamically stable than transition metal oxides [39,41,47], which usually occur on the stainless steels’ and special alloys’ surfaces in high-temperature oxidation (such as Cr_2_O_3_, NiO, Ni(Cr,Al)_2_O_4_, etc.) [52,53]. In our case, thermodynamically stable aluminide layers are covered with another thermodynamically stable Al_2_O_3_ film well bonded to “original” aluminides. The absence of the spallation issue for this type of material corresponds well with other high-temperature oxidation studies [31,33,35,36,37,38].

The proposed thermal diffusion technology can be applied on different components of piping systems, e.g., straight pipes and tubes with lengths up to 10 m, elbows, different connectors, nozzles, and ferrules, particularly for the inner surface components’ protection, which is proven by ETI [29,30,35]. This technology is applicable for different steels and ferrous and Ni-based alloys. Because of this and due to the components’ performance being defined, in a high extent, by the composition and coating structure, there is a good possibility to use this protection route for steels of lower grades, where the products with lower costs would have high performance. Aluminized carbon steel with a very satisfactory performance, as obtained in the present study, is a good example to combine the products’ adequate performance and a competitive cost.

## 4. Conclusions

1. The aluminized and boronized steels significantly outperformed bare stainless steel 316L in high-temperature (500 °C) sulfidation-oxidation conditions. While stainless steel experienced formation of the rather porous sulfide and oxide scale, which are easily detached and peeled-off and which cannot be considered as protective, no sulfide scale formation, blistering and detachment issues, microstructural changes or case depth reduction were observed for the proposed coatings. All studied materials experienced less surface degradation in high-temperature H_2_S–air exposure compared to H_2_S–Ar exposure. The aluminide-based protective layers (on both carbon steel and stainless steel) experienced very insignificant mass gain after sulfidation-oxidation related to surface oxidation with only minimal sulfur presence (below 2 wt.%.) and demonstrated the highest integrity.

2. The enhanced resistance of the proposed materials obtained through the thermal diffusion process is related to the high crystalline lattice energies of borides and aluminides and strong covalent Me-B and Me-Al bonds, which define the high chemical inertness of these compounds. The multi-layered structure consisting of, at least, two inert and well-consolidated protective layers with strong diffusion-induced bonding between the layers and the substrate delays micro-crack propagation, and it is the major structural factor defining the materials’ performance. The thin chemically inert Al_2_O_3_ film occurred on the aluminides’ surface due to high-temperature oxidation, which adhered well to the aluminide surface, promoting corrosion protection of this type of material.

3. The aluminizing process, which can be applied for inner or inner and outer surfaces of tubing and other components made of steels, e.g., stainless steels or even low-cost carbon steels, can be considered as the most reliable for high-temperature sulfidation and sulfidation-oxidation conditions.

4. Since high-temperature processes in the H_2_S-air gas flow significantly differentiate from processes occurring in the H_2_S-Ar environment, and related surface transformations could also be different in these conditions, the present work supplements our studies conducted in H_2_S-Ar environments, providing a better understanding of the processes in high-temperature H_2_S-containing environments. The proposed protection routes of steel components, including long tubing, and employing thermal diffusion technology could be used for further long-term testing and implemented to reduce corrosion issues in service in combustion conditions.

## Figures and Tables

**Table 1:** Chemical composition of steels selected in the work (wt.%).

Substrate	C	Mn	Si	Cr	Ni	Mo	Cu	P	S	Fe *
316L	0.025	1.34	0.50	16.54	10.09	2.02	0.39	0.028	0.004	Bal.
A36/44W	0.18	0.64	0.20	0.10	0.11	0.03	0.31	0.01	0.025	Bal.

* Fe—balance.

**Table 2:** Materials prepared for testing.

Sample Code	Material Description
6	Stainless steel 316L (bare steel)
A	Aluminized carbon steel
6A	Aluminized stainless steel (316)
B	Boronized carbon steel
BT	Boronized carbon steel with additional thin TiO_2_ layer (over the boronized coating)

**Table 3:** Selective EDS analysis data (wt.%) from the surfaces exposed to H_2_S–air at 500 °C for 504 h.

Material	Fe	Cr	Ni	Al	Ti	B	O	S
6—top 6—underneath	20–23 26–28	0.25–0.7 11–12	3–3.5 5–6	- -	- -	- -	50–53 40–45	14–16 8–10
B	38–40	-	-	-	-	2.5–3	44–46	9–10
BT	22–23	-	-	-	6–7	4–4.5	52–54	9–10
A	27–29	-	-	35–37	-	-	29–31.5	1–1.5
6A	25–27	15–16	3.5–4	19–22	-	-	28–30.5	0.7–1

**Table 4:** Assumption of chemical reaction in the present work.

3H_2_S + 3/2O_2_ → 2H_2_S + SO_2_ + H_2_O	1
Fe + xH_2_S → FeS_x_ + xH_2_	2
Fe + S_x_ → FeS_x_	3
FeS + 1/2S_2_ → FeS_2_	4
FeS + H_2_S → FeS_2_ + H_2_	5
2Cr + 3H_2_S → Cr_2_S_3_ + 3H_2_	6
2Cr + 3S_2_ → 2Cr_2_S_3_	7
Ni + H_2_S → NiS + H_2_	8
4FeS_2_ + 11O_2_ → 2Fe_2_O_3_ + 8SO_2_	9
4FeS + 7O_2_ → 2Fe_2_O_3_ + 4SO_2_	0
Ni + 1/2S_2_ → NiS	11
2nFe + SO_2_ → 2Fe_n_O + 1/2S_2_	12
2Fe_3_O_4_ + 1/2SO_2_ → 3Fe_2_O_3_ + 1/4S_2_	13
3Fe + 2O_2_ → Fe_3_O_4_	14
2Fe_3_O_4_ + 1/2O_2_ → 3Fe_2_O_3_	15
2Cr + 3/2O_2_ → Cr_2_O_3_	16
Ni + 1/2O_2_ → NiO	17
Fe + 2Cr + 2O_2_ → FeCr_2_O_4_	18
(2m + 1)Fe + SO_2_ → 2Fe_m_O + FeS	19

**Table 5:** Micro-hardness data (HK0.1) of the studied materials.

Material	Protective Layer/Zone	HK0.1 kgf/mm^2^ Prior Test	HK0.1 kgf/mm^2^ 504h Exposure -Week Testing
Steel (6)	-	260+/−10	205+/−10
B	Top FeB Bottom Fe_2_B Substrate CS	1775+/−50 1625+/−25 180+/−10	1770+/−50 1625+/−25 180+/−10
BT	TiO_2_ FeB Fe_2_B Substrate CS	Could not be determined 1775+/−50 1625+/−25 180+/−10	Could not be determined 1765+/−50 1625+/−25 180+/−10
A	Al-rich layer Main layer (FeAl) Transition (Fe_3_Al) Substrate CS	740+/−25 610+/−25 400+/−25 180+/−10	730+/−25 610+/−25 400+/−25 180+/−10
6A	Al-rich layer Main layer (FeCrNiAl) Transition Substrate 316L	750+/−25 615+/−25 400+/−25 240+/−10	745+/−25 615+/−25 400+/−25 240+/−10

## Data Availability

Data sharing is not applicable.
